# Association Between DOAC Exposure and Lower-Extremity Arterial Calcification: A Propensity-Matched Exploratory CT Study

**DOI:** 10.3390/jcm15093399

**Published:** 2026-04-29

**Authors:** Eniko Pomozi, Dora Zoe Zatyko, Ferenc Imre Suhai, Zoltan Szeberin

**Affiliations:** 1 Department of Interventional Radiology, Heart and Vascular Center, Semmelweis University, 1122 Budapest, Hungary; 2 Heart and Vascular Center, Semmelweis University, 1122 Budapest, Hungary; 3 Department of Vascular and Endovascular Surgery, Heart and Vascular Center, Semmelweis University, 1122 Budapest, Hungary; 4 Department of Cardiology, Heart and Vascular Center, Semmelweis University, 1122 Budapest, Hungary

**Keywords:** peripheral artery disease, vascular calcification, direct oral anticoagulants (DOACs), lower limb arterial calcium score (LLAC), computed tomography angiography (CTA)

## Abstract

**Background**: Lower limb arterial calcification (LLAC) is a robust imaging biomarker of peripheral artery disease (PAD) severity. Vitamin K antagonists are presumed to accelerate cardiovascular calcification. Direct oral anticoagulants (DOACs) may influence vascular calcification differently, but lower limb data are limited. **Methods**: We performed a single-center retrospective cross-sectional study comparing LLAC on clinically acquired non-contrast CT between DOAC users and controls without anticoagulation. Patients were propensity score-matched 1:1 (48 DOAC vs. 48 control; *n* = 96) using baseline clinical covariates. Associations between LLAC scores and perioperative or cardiovascular events were assessed. Segment-specific LLAC was quantified on non-contrast CT and normalized for arterial segment length. A prespecified exposure–duration sensitivity analysis compared the outcomes in patients with ≥5 years of continuous DOAC therapy (*n* = 22) versus matched controls. **Results**: In the matched cohort, total LLAC scores did not differ significantly between DOAC and control groups (infrarenal aorta: median 7596.0 vs. 8637.0 (*p* = 0.487), iliac segment: median 5689.5 vs. 5193.5 (*p* = 0.602). However, in patients with ≥5 years of DOAC use, LLAC scores were significantly lower in proximal segments: infrarenal aorta median 5593.5 vs. 11,185.0 (*p* = 0.001997) and iliac arteries 5624.5 vs. 11,501.0 (*p* = 0.001867)). Higher LLAC was associated with major adverse cardiovascular events (such as myocardial infarction, stroke, or significant bleeding) in controls (*p* = 0.0023) but not in DOAC-treated patients. **Conclusions**: In this propensity-matched, cross-sectional CT study, long-term DOAC exposure was associated with lower proximal LLAC scores in a small duration-defined subgroup, while the primary matched analysis showed no overall difference in total LLAC scores. Because baseline (pre-DOAC) imaging was unavailable and residual confounding/survivor bias is possible, these findings should be considered hypothesis-generating and require prospective validation. The cohort reflected a mixed lower-extremity vascular population rather than exclusively classic chronic atherosclerotic PAD, which may limit biological interpretation and generalizability.

## 1. Introduction

Lower limb peripheral artery disease (LEAD) is a progressive vascular condition characterized by narrowing or occlusion of the lower-extremity arteries, affecting a large proportion of the global population over the age of 65 and contributing substantially to cardiovascular morbidity and mortality [1]. The contemporary 2024 ACC/AHA multisociety guideline further emphasizes that LEAD is not only a limb disease but also a manifestation of systemic atherosclerosis associated with major adverse cardiovascular and limb events, highlighting the importance of improved risk stratification in this population [2]. One of the most robust imaging biomarkers of disease severity and risk of future adverse events in LEAD is vascular calcification. To quantify and measure the severity of calcification, the lower limb arterial calcification (LLAC) score using non-contrast computed tomography (CT) is used [3,4]. Extensive calcification not only reflects advanced disease but also predicts lower limb events, such as reintervention, amputation, and mortality [5,6]. Importantly, lower limb arterial calcification is not a uniform entity. Recent reviews have emphasized that calcification in the lower-extremity arterial tree may occur in both the intimal and medial layers, with distinct biological and clinical implications depending on arterial territory, comorbidity profile, and underlying disease mechanisms [7,8].

Anticoagulant therapy is frequently indicated in LEAD patients due to common comorbidities, including atrial fibrillation (AF), venous thromboembolism (VTE), or mechanical heart valves.

Historical studies have shown that vitamin K antagonists (VKAs), such as warfarin, may accelerate vascular calcification by inhibiting vitamin K-dependent activation of matrix Gla protein (MGP), a potent inhibitor of arterial mineralization [9,10]. This raises concerns that VKA therapy may potentiate disease progression in PAD by exacerbating calcification and increasing the risk of adverse events.

In contrast, direct oral anticoagulants (DOACs), including factor Xa inhibitors (e.g., apixaban, rivaroxaban, edoxaban), and direct thrombin inhibitors (e.g., dabigatran) do not knowingly interfere with vitamin K metabolism [11]. Preclinical and emerging clinical evidence suggest that DOACs may not have the same pro-calcific effects as VKAs and may even play a protective role in terms of worsening vascular calcification [11,12,13]. As an example, rivaroxaban has been shown to reduce expression of pro-calcific markers in vascular smooth muscle cells under uremic and inflammatory conditions, and early observational data suggest slower progression of vascular calcification in DOAC-treated cohorts compared to that on VKAs [14]. Another comprehensive review by Elango et al. further highlighted that multiple retrospective and randomized studies consistently demonstrate a reduced progression of systemic vascular and valvular calcification among DOAC users compared to warfarin, underscoring the potential protective effect through pathways independent of vitamin K metabolism [15]. More recent clinical and translational literature continues to support the concept that DOACs differ from VKAs with respect to vascular calcification biology, although the strength of evidence remains greater in coronary, valvular, and systemic vascular beds than in the lower-extremity circulation [15,16]. However, these findings have been largely extrapolated from coronary vascular beds, with limited data specifically addressing the lower limb vasculature [11,17].

Despite this, no imaging-based study has comprehensively assessed whether DOAC use is associated with differential calcification burden in the lower-extremity arteries among LEAD patients. Given the segment-specific nature of calcification and its prognostic relevance, understanding whether DOAC therapy modulates the extent and distribution of vascular calcification could have important implications for clinical management and secondary prevention strategies. This question may be especially relevant because proximal and distal lower-extremity segments may differ not only in plaque burden but also in the relative contribution of intimal atherosclerotic calcification versus medial arterial calcification, potentially influencing whether any vascular effect of anticoagulant therapy would be uniform across the arterial tree [7,8].

In this study, we aimed to compare the LLAC scores across multiple lower limb arterial segments in LEAD patients receiving DOACs versus matched controls without anticoagulation. Our aim was to fill a crucial gap by providing targeted vascular imaging data on the degree of vascular calcification in anticoagulated PAD patients. Our hypothesis is that DOAC use may be associated with lower LLAC scores, potentially indicating a safer vascular profile than traditional anticoagulants.

## 2. Materials and Methods

### 2.1. Study Design and Objectives

This is a single-center, dual-component study conducted at a tertiary vascular center, comprising: 1. a cross-sectional analysis, evaluating and comparing LLAC scores at a single time point between patients receiving long-term (at least 12 months) direct oral anticoagulant (DOAC) therapy and matched controls without anticoagulation; and 2. a longitudinal observational follow-up, assessing clinical outcomes over time after the index CT imaging, including disease progression, therapeutic interventions, and major adverse limb (revascularization procedure, amputation), cardiovascular events (myocardial infarction, stroke), and mortality. The study was approved by the institutional ethics board (Regional and Institutional Committee of Science and Research Ethics of Semmelweis University (approval number: 9/2023)), and all data were de-identified prior to analysis. The requirement for informed consent was waived due to the retrospective nature of the study and the use of de-identified data.

**Figure 1:**
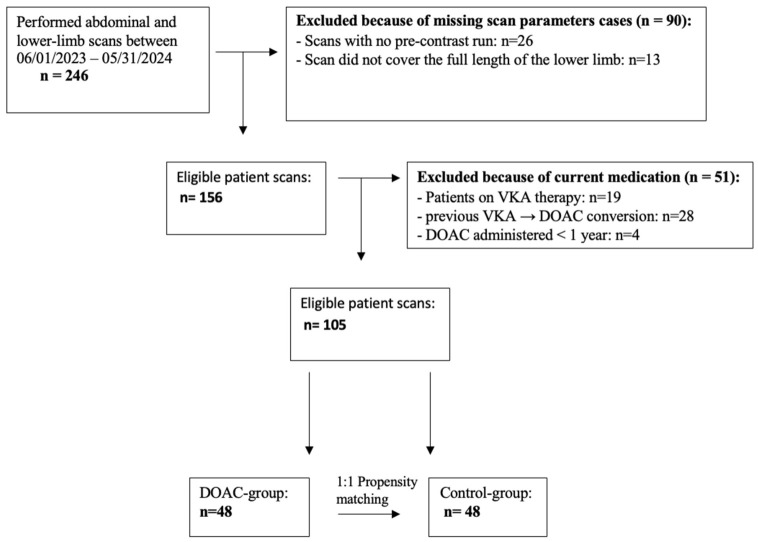
Flowchart of the patient inclusion/exclusion process. DOAC—direct oral anticoagulant, VKA—vitamin K antagonist.

The whole patient exclusion–inclusion process is visualized in Figure 1.

The study initially aimed to compare the impact of long-term VKAs versus DOAC therapy on lower limb arterial calcification among patients with chronic lower-extremity peripheral artery disease. However, due to the widespread clinical transition from VKA to DOACs, the number of eligible patients on sustained VKA therapy during the 12-month data collection period was insufficient for meaningful statistical comparison. Consequently, the study design was revised to compare LLAC scores between patients on long-term DOAC therapy and matched controls not receiving any anticoagulation.

Also, although the original aim was to focus exclusively on chronic LEAD patients, the eligible cohort was expanded to include individuals undergoing lower limb CT angiography for a variety of clinical indications, such as acute-on-chronic limb ischemia (purely acute limb ischemia cases without history of PAD and any calcification on the CT-scan were excluded) or non-occlusive conditions such as popliteal artery aneurysms and other preoperative vascular evaluations (arteriovenous fistula or iatrogenic pseudoaneurysm). This broader inclusion strategy was considered necessary to preserve an analyzable matched imaging cohort; however, it also means that the observed LLAC patterns should be interpreted within a mixed lower-extremity vascular population rather than as specific to classic chronic atherosclerotic PAD alone. While not all subjects had classic atherosclerotic LEAD, all patients had a clear indication for lower limb vascular imaging and collectively represent the clinical spectrum of LEAD. The inclusion of this broader patient population was necessary to ensure adequate statistical power and reflects the real-world distribution of vascular pathologies encountered in contemporary vascular imaging practice.

### 2.2. Propensity Score Matching

**Figure 2:**
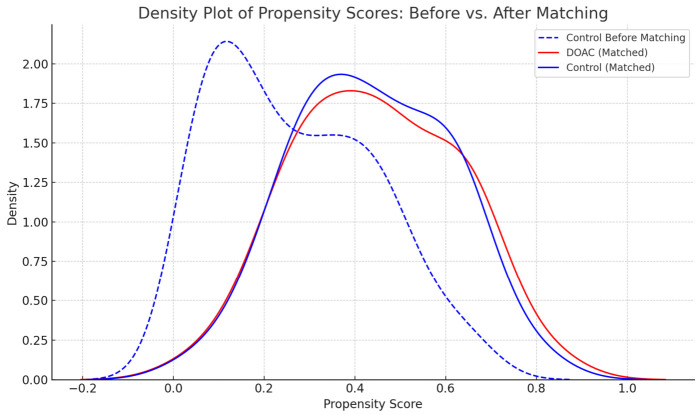
Density plot of propensity scores: dashed blue line—control population before matching (dashed blue): shows wide, unbalanced distribution; solid red line—DOAC group; solid blue line—control group (propensity-matched): closely overlaps DOAC group after matching.

To reduce confounding related to differences in clinical characteristics between anticoagulated and non-anticoagulated patients, we used propensity score matching to create comparable cohorts. Propensity scores were estimated using a logistic regression model, with DOAC exposure as the dependent variable and baseline covariates selected a priori based on clinical relevance and availability, including age, sex, comorbidities (including as diabetes mellitus and chronic kidney disease), and the clinical indication for vascular imaging, disease severity, and prior interventions. In patients with chronic PAD, disease severity was assessed using the Rutherford classification, whereas in patients with other vascular indications, severity was characterized using a predefined symptom-based scoring system. A total of 48 DOAC-treated patients were matched to 48 non-anticoagulated control patients based on these scores. The distribution of propensity scores before and after matching is shown in Figure 2.

Patients were matched 1:1 without replacement using nearest-neighbor matching on the logit of the propensity score with a caliper of 0.2 standard deviations of the logit propensity score. Matching performance was assessed using standardized mean differences (SMDs) for each covariate before and after matching; SMD < 0.10 was considered to indicate acceptable balance. We additionally inspected propensity score overlap using density plots and histograms to confirm adequate common support. This process yielded 48 DOAC patients matched to 48 controls (*n* = 96). For the duration-based sensitivity analysis, we evaluated the subset of DOAC patients with ≥5 years of continuous therapy (*n* = 22) and their matched controls; baseline characteristics of this subgroup were reported separately to assess potential differences from the overall matched cohort.

### 2.3. Imaging Parameters and LLAC Scoring

All patients underwent lower-extremity computed tomography angiography (CTA) between 1 June 2023 and 31 May 2024, using a third-generation dual-source CT scanner (Somatom Force, Siemens Healthineers, Forchheim, Germany). A dedicated acquisition protocol was applied, consisting of an unenhanced (native) scan followed by a dual-energy contrast-enhanced runoff scan. Scans were acquired in helical mode from craniocaudal direction using automatic tube current modulation (CARE Dose4D), with dual-energy acquisition at 80 kV and Sn150 kV.

Native images were acquired with a collimation of 192 × 0.6 mm, pitch 0.6–0.7, and rotation time between 0.25 and 0.5 s. The images were reconstructed at 3.0 mm slice thickness using an increment of 2.0–3.0 mm, applying the Bv36 kernel with ADMIRE iterative reconstruction. The field of view was 380 mm, with coverage extending from the infrarenal aorta to the ankles (approximately −6.5 mm to 1393 mm), encompassing the full length of the lower limb arterial system.

**Figure 3:**
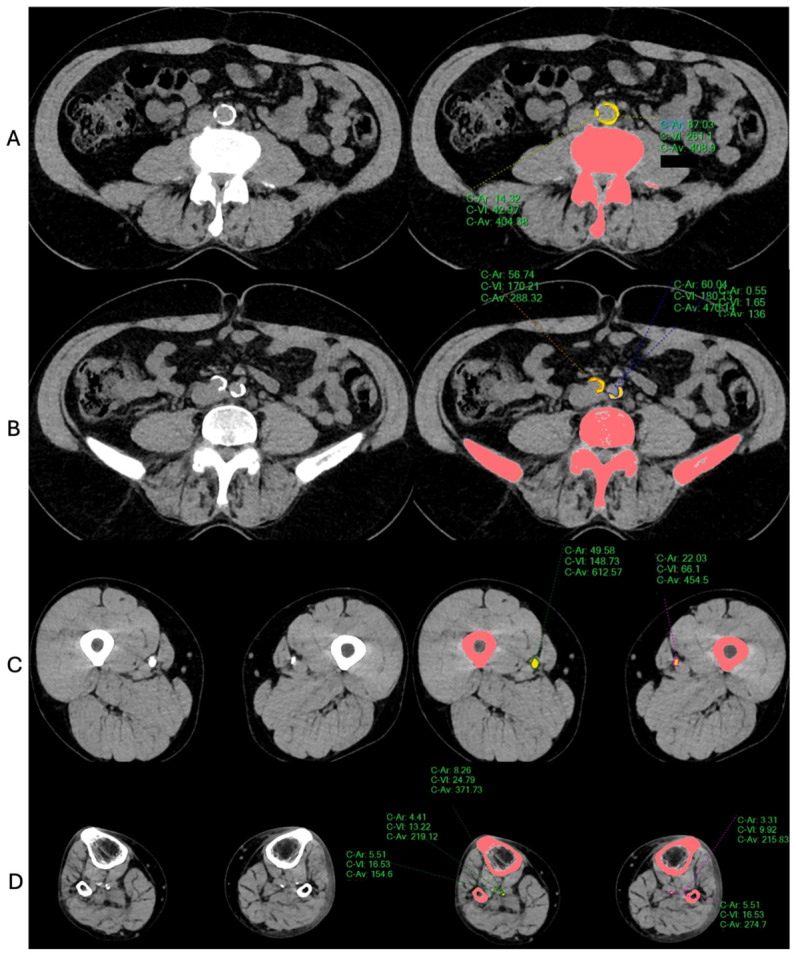
Calcification measurement using the integrated ‘Calcium Scoring’ tool of the software ‘Philips IntelliSpace Portal’ at the (**A**) infrarenal aorta, (**B**) iliac bifurcation, (**C**) superficial femoral, and (**D**) crural segments. Structures highlighted in pink color = automatically segmented bone generated by the Philips IntelliSpace Portal software’s calcium scoring tool, yellow markings = manually segmented arterial calcifications.

Vascular calcification analysis was performed using the non-contrast dataset. Image processing and quantification were carried out on the Philips IntelliSpace Portal, version 12.1.10 (Philips Healthcare, Best, The Netherlands) using the integrated ‘Calcium Scoring’ tool; this process is shown in Figure 3. For calcification evaluation, we followed the method prescribed by Chowdhury et al. [18] in their publication, based on the Agaston scoring method [19]: calcified lesions were defined as ≥130 Hounsfield units (HU) in at least 3 contiguous voxels. A modified Agatston method was used, where the lesion area was multiplied by a weighting factor based on peak attenuation: 130–199 HU = 1, 200–299 HU = 2, 300–399 HU = 3, and ≥400 HU = 4. The scores were summed per axial slice and across the full length of each arterial segment to calculate segmental LLAC values. Final LLAC was reported as the total raw score across all analyzed segments of both legs.

For this study, the lower-extremity arterial tree was defined from the infrarenal aorta down to the ankles in both legs. The vasculature was segmented anatomically into three regions: (1) the infrarenal-aortic (A) segment, extending from the lowermost renal artery to the iliac bifurcation; (2) iliac segment, extending from the bifurcation to the distal external iliac arteries; (3) the femoro-popliteal (FP) segment, covering the region from the common femoral artery to the below-knee popliteal artery; and (4) the crural segment, including the tibio-peroneal trunk and the anterior, posterior tibial, and peroneal arteries down to the level of the ankle joint.

All image analyses were performed by a radiology resident (E.P.) with prior clinical experience in vascular surgery. Selected cases were additionally reviewed in collaboration with a senior vascular surgery resident (Z.D.). The process was supervised by a board-certified radiologist (F.S.) with subspecialty expertise in cardiovascular imaging. A standardized workflow within the IntelliSpace platform was used for all measurements. Formal inter-reader and intra-reader reproducibility analyses were not prespecified for this retrospective study and are therefore unavailable.

### 2.4. Statistical Analysis

Basic descriptive statistics were calculated for all relevant variables. Categorical variables were summarized as frequencies and percentages, and continuous variables were summarized using means and standard deviations (for normally distributed data) or medians and interquartile ranges (for non-normally distributed data).

Normality of continuous variables was assessed using the Shapiro–Wilk test.

Due to variable image quality and anatomical coverage, LLAC scores were not obtainable for all patients in every arterial segment. Segment-wise analyses were therefore conducted using all available data per segment, with sample sizes ranging from 40 to 47 patients in the DOAC group and from 42 to 48 in the control group.

To accommodate partially missing data and maintain methodological consistency, a normalized per-patient value was calculated for each segment. If bilateral LLAC measurements (left and right) were available, the values were averaged to obtain a single representative score. If only one side was available, that value was used directly. This approach ensured comparability across patients and reduced bias that could arise from summing bilateral data or excluding partially complete cases. No systematic differences were observed in median LLAC values between patients with bilateral and unilateral data for any segment, indicating that the side-averaging approach and inclusion of partial data did not bias the results. This supports the robustness of the final per-patient segment scores and group comparisons.

To compare categorical clinical outcomes, including treatment modalities, reoperation rates, amputation incidence, bleeding events, and mortality between the DOAC and control groups, we performed chi-square tests of independence; when expected cell counts were fewer than five, Fisher’s exact test was applied to ensure statistical validity.

All statistical analyses were conducted using IBM SPSS Statistics (version 29.0.1; IBM Corp., Armonk, NY, USA). A two-sided *p*-value < 0.05 was considered statistically significant.

#### 2.4.1. Evaluation of the Difference in LLAC Scores Between DOAC and Control Groups

During the comparison of the LLAC scores between groups, continuous variables were summarized as median (IQR) due to non-normal distributions (assessed using the Shapiro–Wilk test). Because patients were matched 1:1, primary between-group comparisons were performed within matched pairs using the two-sided Wilcoxon signed-rank test on the paired difference (DOAC − control). The analyses were conducted for raw LLAC and stratified by arterial segment (infrarenal aorta, total iliac, total femoro-popliteal, and total crural).

To evaluate whether the association between DOAC exposure and LLAC differed by treatment duration, DOAC-treated patients were stratified a priori by years on anticoagulation into 1–2 years, 3–4 years, and ≥5 years. Within each duration stratum, LLAC in DOAC users was compared to their corresponding propensity-matched controls using two-sided Wilcoxon signed-rank tests (paired non-parametric testing), applied separately for the LLAC scores in each anatomical segment (infrarenal aorta, total iliac, total femoro-popliteal, and total crural) and for total LLAC where applicable. The analyses were performed on a complete-case basis per segment, including only matched pairs with available LLAC measurements for both members of the pair.

#### 2.4.2. Evaluation of the Difference Between Follow-Up Events Linked to LLAC Scores Between DOAC and Control Groups

The association between total LLAC scores and type of revascularization strategy (medical therapy, endovascular, open surgery, or hybrid procedure) was assessed using the non-parametric Kruskal–Wallis test. For binary outcomes, including the presence of reoperations, amputations, major adverse events (myocardial infarction, ischemic stroke, hemorrhagic stroke, or significant bleeding), and mortality, the total LLAC scores were compared between patients with and without each outcome using the Mann–Whitney U test.

Comparisons of categorical event rates (e.g., proportion of patients with reoperations, amputations, major adverse events, or death) between the DOAC and control groups were evaluated using the chi-square test of independence. Fisher’s exact test was used for contingency tables with expected counts less than five. Subgroup analyses were performed descriptively to assess the distribution of bleeding events according to DOAC type and dosing; no formal statistical comparison was conducted due to the small sample sizes within these strata.

## 3. Results

### 3.1. Demographic and Clinical Characteristics

The demographic parameters of the patients are summarized in Table 1: a total of 96 patients were included in the study, with 48 patients in the DOAC group and 48 in the matched control group. The median age was slightly lower in the DOAC group (72.0 years) compared to the control group (74.0 years).

Regarding sex distribution, the DOAC group included 37 males (77%) and 11 females (23%), while the control group included 33 males (69%) and 15 females (31%).

The indications for CT imaging varied slightly between groups. Most scans were performed for chronic LEAD, with 34 cases (71%) in the DOAC group and 40 cases (83%) in the control group. Acute-on-chronic limb ischemia was present in seven DOAC patients and two controls. Aneurysmatic disease of the iliac or popliteal segment and post-procedural complications such as pseudoaneurysm or arteriovenous fistula were observed in both groups at similar frequencies.

The severity of chronic LEAD was assessed using the Rutherford classification. In the DOAC group, most patients fell into Rutherford categories 2 (*n* = 11) and 3 (*n* = 11), while more advanced stages (Rutherford 5 and 6) were observed in five patients each. In contrast, the control group exhibited a broader distribution, with 13 patients in category 2, eight in category 3, and 11 patients presenting with Rutherford stage 5.

Comorbidities were prevalent across both groups. Hypertension and hyperlipidemia were the most frequent conditions, affecting over two-thirds of both cohorts. Diabetes mellitus was observed in 25 DOAC patients and 22 controls. Notably, end-stage renal disease (ESRD) was more common in the DOAC group (*n* = 6) than in controls (*n* = 3), and several patients in the DOAC group were on dose-adjusted regimens due to renal impairment. Smoking status was recorded in 23 DOAC patients (data unavailable for 12 cases) and 29 controls (data missing for eight cases).

The use of platelet aggregation inhibitors (PAI) was slightly lower in the DOAC group (*n* = 39) compared to controls (*n* = 46), whereas statin use was equal in both groups (*n* = 39).

Among the DOAC users, apixaban (Eliquis) was the most prescribed agent, with standard dosing (2 × 5 mg) in 15 patients and reduced doses (2 × 2.5 mg or 1 × 2.5 mg) in 12 patients, particularly among those with ESRD. Rivaroxaban (Xarelto) was used in nine patients, dabigatran in eight (including generic Telexer in one), and edoxaban in four.

The primary indication for DOAC therapy was atrial fibrillation (*n* = 39), followed by deep vein thrombosis (*n* = 2), pulmonary embolism (*n* = 2), combined DVT and PE (*n* = 1), and prior stroke (*n* = 1). In three patients, the indication was not documented.

### 3.2. Perioperative and Follow-Up Data

From the time of the CT-scan, the mean follow-up duration was 13.6 ± 8.1 months across the cohort. The perioperative data of the patients is summarized in Table 2. Following imaging, 73% of DOAC patients and 67% of controls underwent an endovascular, open surgical, or hybrid procedure. As also shown in Table 2, no statistically significant differences were observed; in the DOAC group, 14 patients received endovascular treatment, 14 underwent open surgical revascularization, and five were treated with hybrid procedures. Medical therapy alone was pursued in nine patients, while no revascularization option was available for six. In comparison, the control group had a slightly different distribution, with 14 patients undergoing endovascular procedures, nine undergoing open surgery, and nine receiving hybrid interventions, while medial therapy alone was used in 12 cases, and no revascularization was feasible in four patients.

The anatomical regions treated also differed slightly between groups. In the DOAC group, most interventions targeted the femoro-popliteal segment (*n* = 15), with additional procedures involving the iliac (*n* = 9), aorto-iliac (*n* = 2), and crural (*n* = 1) segments, as well as combined territories such as iliac and femoro-popliteal (*n* = 4), or femoro-popliteal and crural (*n* = 2). In the control group, femoro-popliteal involvement was also most common (*n* = 13), with frequent combined iliac and femoro-popliteal interventions (*n* = 9), isolated iliac (*n* = 7), aorto-iliac (*n* = 1), aorto-iliac + femoro-popliteal (*n* = 1), and femoro-popliteal + crural (*n* = 1) treatments. In total, the femoro-popliteal segment was the most frequently treated region in both cohorts.

Reoperations were required in 10 patients (13 procedures) in the DOAC group and in 11 patients (16 procedures) in the control group. Most DOAC patients underwent a single reoperation (*n* = 8), while two patients required two or more reinterventions. In the control group, six patients had one reoperation, and five patients had two. Reinterventions occurred more frequently beyond 30 days in the control group (*n* = 12 vs. *n* = 4), whereas the DOAC group experienced earlier (<30 day) reoperations (*n* = 9), including three within 24 h (two due to bleeding and one due to early reocclusion). Common causes of reoperation in both groups included restenosis/reocclusion (DOAC: *n* = 5; control: *n* = 8), septic bleeding (*n* = 2 in each group), and lymphatic complications (*n* = 2 in each group).

Amputation rates were equal between groups (*n* = 8 in each). However, the timing and level of amputation differed. The DOAC group had more early amputations (<30 days: *n* = 5), while the control group had more delayed amputations (five between 30 days and 6 months). Most amputations in the DOAC group were at the femoral level (*n* = 6), whereas the control group had more distal amputations, with five cases below the ankle.

Cardiovascular complications were similar in the control group, with six cases of acute myocardial infarction (vs. 4 in DOAC group). Both groups had one ischemic stroke each. Bleeding events were notably higher in the DOAC group, including five gastrointestinal bleeds, two nasal bleeds, and one hemorrhagic stroke, while no bleeding events were reported in the control group.

The mortality rate was slightly higher in the DOAC group (*n* = 9) compared to the control group (*n* = 6). In the DOAC cohort, causes of death included cardiovascular events (*n* = 2), sepsis from limb wounds (*n* = 4), stroke (*n* = 1), a perforated cholecystitis (*n* = 1), and one case of suicide. In the control group, deaths were attributed to cardiovascular causes (*n* = 2), metastatic cancer complications (*n* = 1), pneumonia (*n* = 1), sepsis (*n* = 1), and stroke (*n* = 1).

As shown in Table 2, no statistically significant differences were observed between the DOAC and control groups across secondary outcomes, including reoperations, amputations, bleeding events, and mortality (all *p* > 0.05).

### 3.3. Comparison of LLAC Scores Between DOAC and Control Groups

When comparing the entire DOAC cohort to the control group, there were no statistically significant differences in the LLAC scores for any segment (Table 3). The median LLAC for the infrarenal aorta was 7596.0 [IQR: 5063.0–11,712.0] in the DOAC group and 8637.0 [IQR: 5626.0–12,241.0] in the control group (*p* = 0.487). Similar results were observed for the total iliac (5689.5 vs. 5193.5; *p* = 0.602), femoro-popliteal (6089.0 vs. 6582.0; *p* = 0.789), and crural segments (1349.5 vs. 1431.5; *p* = 0.390) (Table 3). These findings indicate that, using the LLAC scores, DOAC therapy was not associated with significant differences in overall calcification scores compared to controls.

### 3.4. DOAC Duration-Stratified Exploratory Analysis

To preserve the matched-pair structure, duration-stratified comparisons of LLAC scores between DOAC users and their matched controls were performed using two-sided paired Wilcoxon signed-rank tests within three prespecified DOAC exposure strata (1–2 years, 3–4 years, and ≥5 years). In the 1–2 year stratum (*n* = 12 DOAC vs. *n* = 12 controls), no statistically significant differences were observed across segments: infrarenal aorta (median 8838.5 [IQR 5219.2–12,423.0] vs. 6098.5 [3960.8–11,682.2]; *p* = 0.312), total iliac (13,582.5 [4801.5–15,631.5] vs. 8006.0 [5706.2–10,625.8]; *p* = 0.260), femoro-popliteal total (16,613.5 [9789.8–29,065.8] vs. 9308.5 [7470.0–13,373.8]; *p* = 0.126), crural total (3782.5 [1162.0–7828.2] vs. 2024.5 [1438.5–3628.2]; *p* = 0.707), and total LLAC (44,068.5 [31,138.2–69,837.0] vs. 27,772.5 [18,475.2–33,825.0]; *p* = 0.089).

In the 3–4 year stratum (*n* = 13–14 per segment; see Table 4), differences again did not reach statistical significance: infrarenal aorta (10,570.0 [7475.0–14,353.0] vs. 8637.0 [4932.0–10,525.0]; *p* = 0.209), total iliac (10,905.5 [6850.2–14,778.2] vs. 7524.5 [4570.2–16,567.0]; *p* = 0.194), femoro-popliteal total (11,515.0 [710.8–23,424.8] vs. 5888.5 [2897.0–17,440.8]; *p* = 0.626), crural total (4030.0 [1176.0–6106.2] vs. 2554.5 [1180.5–8096.2]; *p* = 0.855), and total LLAC (43,000.5 [24,334.0–57,192.5] vs. 21,478.0 [15,196.2–53,206.0]; *p* = 0.296).

**Figure 4:**
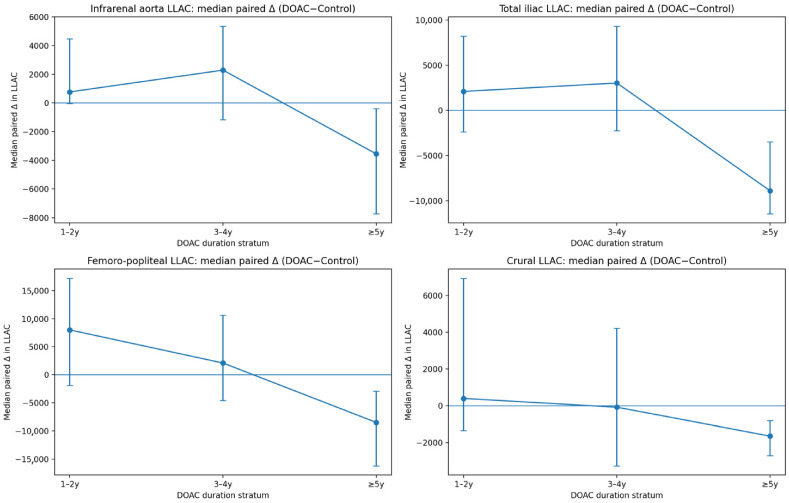
Duration-stratified matched-pair differences in LLAC (DOAC − matched control) with uncertainty estimates. Panels show the median paired difference in LLAC between DOAC-treated patients and their propensity-matched controls (ΔLLAC = DOAC − control) across DOAC exposure strata (1–2 years, 3–4 years, ≥5 years) for the infrarenal aorta, total iliac, total femoro-popliteal, and total crural segments. Points represent the median ΔLLAC within each stratum, and error bars denote bootstrap 95% confidence intervals. The horizontal line at 0 indicates no difference between matched groups (values below 0 indicate lower LLAC in DOAC patients relative to their matched controls).

In the ≥5 year stratum (*n* = 20–22 per segment), the DOAC users had lower LLAC scores in proximal segments, which appeared to be statistically significant: infrarenal aorta (5593.5 [4521.2–8565.8] vs. 11,185.0 [6973.0–14,395.0]; *p* = 0.001997), total iliac (5624.5 [3012.0–7812.5] vs. 11,501.0 [8684.8–19,352.8]; *p* = 0.001867), and total LLAC (23,847.0 [17,446.8–36,968.0] vs. 49,213.5 [30,523.5–60,363.0]; *p* = 0.001355). The differences in femoro-popliteal and crural segments trended in the same direction but did not meet conventional statistical significance (femoro-popliteal: 7809.0 [4565.2–12,814.0] vs. 18,827.0 [10,591.2–22,566.2]; *p* = 0.0557; crural: 1839.5 [990.8–5612.8] vs. 4793.5 [1944.5–8414.2]; *p* = 0.0588). Given the modest sample sizes within strata and the multiple segment-level comparisons performed, these duration-stratified findings should be interpreted with caution and viewed as exploratory and hypothesis-generating rather than as evidence of a definitive duration-dependent association. The corresponding duration-stratified LLAC results are presented in Table 4, and matched-pair median differences across strata are illustrated in Figure 4.

### 3.5. Association of LLAC Scores with Clinical Outcomes

Reoperations were required in 21% of DOAC patients and 23% of controls, with no significant difference in reoperation rates between groups (*p* = 1.0). Total amputation rates were also comparable (DOAC 15% vs. control 13%, *p* = 1.0). Major adverse cardiovascular or bleeding events occurred in 27% of the DOAC group and 17% of controls (*p* = 0.32), while mortality rates were 19% and 13%, respectively (*p* = 0.57).

When comparing total LLAC scores across different revascularization strategies, no significant differences were observed in either group (DOAC *p* = 0.16; control *p* = 0.87, Kruskal–Wallis test). There was also no significant association between total LLAC scores and the need for reoperations (DOAC *p* = 0.51; control *p* = 0.45), amputations (DOAC *p* = 0.73; control *p* = 0.13), or mortality (DOAC *p* = 0.40; control *p* = 0.16).

Interestingly, in the control group, the patients who experienced major adverse events such as myocardial infarction, stroke, or significant bleeding had significantly higher total LLAC scores compared to those without events (*p* = 0.0023). This relationship was not observed in the DOAC group (*p* = 0.17).

### 3.6. Bleeding Events Among DOAC-Treated Patients

Within the DOAC group, seven patients experienced gastrointestinal (GI)- or nasal bleeding. There was no significant difference in the total LLAC scores between patients with and without these bleeding events (*p* = 0.26). Descriptive subgroup analysis showed that bleeding events occurred primarily among patients receiving standard-dose rivaroxaban (Xarelto) and apixaban (Eliquis). However, the small numbers preclude definitive conclusions regarding the influence of DOAC type or dosing on bleeding risk.

## 4. Discussion

In this matched cohort analysis, we explored whether lower limb arterial calcification (LLAC) scores differ between patients with LEAD receiving DOAC therapy and matched non-anticoagulated controls, and whether LLAC relates to perioperative and follow-up outcomes. Overall, pooled analyses across all DOAC durations did not demonstrate significant differences in raw LLAC between DOAC users and controls. However, exploratory duration-stratified analyses suggested a duration-dependent pattern, in which a consistent reduction in LLAC emerged only among patients receiving DOACs for ≥5 years. Given the retrospective cross-sectional design and modest subgroup sizes, these findings should be interpreted as hypothesis-generating and primarily useful for guiding future prospective study design rather than supporting causal inference.

An additional point relevant to interpretation is that the study cohort was not restricted to classic chronic PAD alone, but reflected a broader lower-extremity CTA population that also included acute-on-chronic ischemia and selected non-occlusive vascular conditions. While this improves real-world clinical applicability, it also introduces heterogeneity in underlying disease biology and calcification phenotype. Accordingly, the segment-level LLAC patterns observed in this study should be interpreted as reflecting a mixed lower-extremity vascular population rather than a purely chronic atherosclerotic PAD cohort.

### 4.1. Discussing Measurement Variability Across Arterial Segments

The first point that should be considered when interpreting segment-level LLAC findings is the potential for measurement variability across both arterial territories and CT acquisition/reconstruction settings [20,21]. This issue is particularly relevant in lower-extremity imaging, where vessel caliber, wall morphology, and calcification pattern differ substantially from the infrarenal aorta and iliac arteries to the femoro-popliteal and crural beds [21,22]. Distal arteries are smaller, more tortuous, and more susceptible to partial-volume effects, which can make calcium boundaries less stable and amplify the impact of small differences in segmentation or threshold-based detection [20,21,23]. In addition, calcification quantification by CT is sensitive to technical parameters such as slice thickness, reconstruction algorithm/kernel, image noise, and attenuation behavior [20,21,23,24]. In a cadaver leg study specifically addressing lower-extremity calcium scoring, modified Agatston and volume scores were significantly influenced by slice thickness reconstruction and reconstruction method, whereas tube current had less effect, underscoring the need for protocol standardization in leg artery calcium quantification [20]. More broadly, recent reviews of peripheral arterial calcium scoring have emphasized that methodological heterogeneity remains a major limitation of the field and that standardization is needed before scores can be compared confidently across cohorts or platforms [7,22]. Although much of the technical reproducibility literature comes from coronary calcium scoring, the same measurement principles apply: thinner slices may improve small-calcification detection but can also alter score magnitude through changes in noise and partial-volume behavior, and reconstruction choices can shift quantified calcium scores enough to affect reproducibility [25]. Finally, newer peripheral CT studies using photon counting and different vascular kernels further demonstrate that reconstruction settings materially influence image quality and delineation of calcified lower-extremity vessels, especially in small-diameter runoff arteries [23,24]. Accordingly, our territory-specific findings—particularly in distal segments—should be interpreted as reflecting not only possible biological differences in calcification distribution but also the inherent technical variability of CT-based calcium quantification in the lower-extremity arterial tree [7,20,22,23,24,25].

### 4.2. Segment-Specific LLAC Scores: A Differential Effect

One of our key observations is that however LLAC scores of the DOAC group in all arterial segments did not differ from the LLAC values of the control group, in contrast, the ≥5-year DOAC subgroup showed a consistent pattern of lower calcification scores compared with matched controls, particularly in proximal and composite measures (including the iliac segment in paired comparisons). This partially contrasts with the initial hypothesis that DOACs, which do not inhibit vitamin K-dependent activation of matrix Gla protein (MGP), would exert the strongest protective effect on medial arterial calcification (MAC), the dominant phenotype below the knee [25,26]. The observed duration-dependent signal appearing more clearly in proximal segments may reflect differences in underlying calcification biology between vascular territories, but it may also be influenced by residual confounding and selection effects inherent to long-term therapy exposure, and these observations should be interpreted as concept-forming rather than confirmatory.

An important alternative explanation for the lower proximal LLAC observed in the ≥5-year DOAC subgroup is selection/survivor bias. Patients who remain on continuous DOAC therapy for >5 years are likely to represent a selected subgroup with better treatment persistence, greater healthcare engagement, lower frailty, and fewer competing adverse events than the broader LEAD population. This is closely related to the healthy adherer effect, a well-described source of bias in observational studies, and to survivor/immortal time-related bias, whereby patients must survive and remain event-free long enough to enter the longest-exposure category. Therefore, the apparent long-duration association may reflect patient selection and treatment persistence as much as any direct biological effect of DOAC exposure on calcification [27,28].

This unexpected segmental pattern may be in connection with distinct pathophysiological mechanisms. First, the infrarenal aorta and iliac arteries are typically affected by intimal atherosclerotic calcification, driven by lipid accumulation, inflammation, and plaque formation. DOACs, particularly factor Xa inhibitors, like rivaroxaban and apixaban, may reduce thrombin generation and downstream inflammatory cascades that accelerate intimal plaque progression [15,29]. In contrast, MAC is a matrix-driven process involving vascular smooth muscle cell (VSMC) osteogenic transformation and is strongly associated with advanced diabetes mellitus (DM) and chronic kidney disease (CKD) [15,29,30]. Although we hypothesized that DOACs would most significantly affect crural MAC via preserved MGP activity, our inability to detect significant differences in this segment may stem more from technical and sample size limitations than true biological absence of effect.

These findings can also be partially explained by the advanced comorbidity profile of our study population. Most of the patients, including those in the DOAC group, had long-standing diabetes mellitus and/or end-stage renal disease (ESRD), both of which are strongly associated with early-onset and progressive medial artery calcification, particularly in below-the-knee arteries. In such patients, MAC may already be well-established by the time DOAC therapy is initiated, potentially limiting its capacity to stabilize these lesions. The findings by Liu et al. and Meer et al. support the view that advanced MAC is associated with persistent distal ischemia and a higher risk of limb loss despite technically successful revascularization [14,30]. Indeed, as Konijn et al. and Guzman et al. highlight, crural calcification is a potent predictor of major amputation risk in PAD [5,6], and so do the recent findings by Lee et al., who demonstrated that higher tibial and total lower-extremity calcium scores significantly predict amputation risk in patients with PAD [31].

Additionally, DOACs may primarily modulate intimal atherosclerotic calcification through effects on thrombin signaling, pro-inflammatory pathways, and altering endothelial integrity, which are more relevant in proximal large arteries (aorta, iliac, and fem-pop segments). In contrast, MAC is a matrix-driven process that may be less sensitive to anticoagulant modulation once established [15,32].

Another explanation for the proximal-versus-distal divergence is that the absence of a statistically significant crural signal may reflect both biology and measurement limitations, rather than sample size alone. Recent reviews emphasize that MAC is a distinct, highly regulated process associated with vascular smooth muscle cell osteogenic transition, extracellular matrix remodeling, and mineral dysregulation, and it is especially prevalent in small- and medium-caliber arteries of the lower extremities [8]. This suggests that distal crural calcification may be biologically less responsive to thrombin- or factor Xa–pathway modulation than proximal intimal calcification, where coagulation-linked protease signaling is more directly implicated in vascular remodeling and smooth muscle cell phenotype switching [33]. At the same time, distal-segment calcium quantification is technically more challenging: smaller vessel diameter, tortuosity, partial-volume effects, and lower segment evaluability can reduce the stability of CT-based calcium scoring in runoff arteries. Supporting this, lower-extremity CT calcium scoring studies have shown that quantified calcification severity is affected by reconstruction settings such as slice thickness and reconstruction algorithm, and recent systematic reviews highlight persistent methodological heterogeneity across peripheral calcium-scoring studies [7,21,22]. Accordingly, the lack of significance in the crural segment in our cohort should be interpreted cautiously as potentially reflecting a combination of true biological differences between proximal intimal and distal medial calcification, limited statistical power, and greater technical variability in distal CT-based calcium quantification, rather than any single explanation alone.

### 4.3. Implications for Systemic Atherosclerosis and Cardiovascular Risk

There is an emerging amount of evidence that LLAC not only serves as a predictor of local vascular disease but also as an indicator for systemic atherosclerotic burden. Multiple studies, including those by Aly et al., Meer et al., Shin et al. and Zwakenberg et al. [34,35,36,37], all have demonstrated significant correlations between lower-extremity arterial calcification and coronary artery calcification, suggesting a shared systemic pathophysiology. This link also highlights the relevance of our finding that long-term DOAC use was associated with significantly lower LLAC in proximal segments, which may indicate reduced systemic atherosclerotic activity. The large population studies by Aly et al. [34] and Meer et al. [35] suggest that the extent and pattern of lower-extremity calcification can refine risk stratification beyond traditional scores.

In our study, higher LLAC scores correlated with major adverse cardiovascular events (MACE) in the non-anticoagulated control group but not among DOAC users. This observation supports the theory that anticoagulation may modify the pro-thrombotic consequences of heavily calcified plaques. Chang et al. reported an inverse relationship between LLAC scores and acute thrombosis risk, suggesting that high calcific burden may stabilize plaques but increase procedural complexity [38]. Our results expand this view, suggesting that DOACs could cover thrombogenic potential associated with advanced vascular calcification.

### 4.4. Revascularization and Perioperative Outcomes

Our analysis of perioperative and follow-up outcomes complements recent literature, highlighting the predictive value of LLAC scores for procedural planning. High segmental calcium scores, particularly in the iliofemoral arteries, have been linked to higher reintervention, limb loss, and mortality rates after endovascular therapy [39]. Besides that, the work by Devia-Rodriguez et al. and Huynh et al. further suggests that calcium scores derived even from contrast-enhanced CT scans can provide actionable prognostic information [40,41]. In our study, the LLAC score was not significantly associated with reoperation, amputation, or mortality in either cohort, aligning with findings from Huang et al. that procedural outcomes may depend more on technical and hemodynamic factors than calcification alone. However, the significant link with MACE in controls underscores the broader cardiovascular risk that severe LLAC conveys [42].

Bleeding risk in the DOAC group warrants further consideration. Although gastrointestinal bleeding did not differ significantly between groups, it occurred only in the DOAC cohort (5/48, 10.4%) and in none of the matched controls (0/48), with a borderline *p*-value (*p* = 0.056). While this study was not powered for formal bleeding risk comparisons, this numerical imbalance is clinically relevant and should be considered when interpreting the overall risk–benefit profile of long-term DOAC therapy in this lower-extremity arterial disease population. Accordingly, any potential vascular association observed in exploratory imaging analyses should be weighed against the possibility of increased bleeding complications. The descriptive analysis revealed that most bleeding events occurred in patients on standard-dose rivaroxaban and apixaban. This finding aligns with the known bleeding risk profiles of these agents but should be interpreted cautiously due to the small event numbers and dosing heterogeneity, and it raises concerns about the safe adjustment of the DOAC type and doses individually in everyday patient care [43,44,45].

## 5. Limitations

This study has several important limitations that require cautious interpretation. First, the design is retrospective and cross-sectional with respect to calcification assessment; because baseline (pre-DOAC) imaging was not available, the analyses cannot evaluate calcification progression or establish a causal treatment effect. Second, the cohort is modest in size, and the duration-defined subgroups are smaller, increasing susceptibility to unstable effect estimates and type I error, particularly given multiple comparisons across vascular segments. Third, although matched-pair methodology reduces measured baseline imbalance, residual confounding remains possible, including confounding by indication, differences in preventive care intensity (e.g., lipid lowering, antiplatelet strategies), and survivor bias/healthy adherer bias in the ≥5-year exposure group (patients who remain on therapy long-term may differ systematically from those with shorter exposure). In addition, because the cohort included patients with vascular indications beyond classic chronic PAD, including acute-on-chronic ischemia and selected non-occlusive conditions, clinical heterogeneity may also have influenced calcification patterns and limited the interpretation of the findings as specific to chronic PAD alone.

Fourth, the cohort reflects a real-world lower-extremity CTA population and therefore included patients with vascular indications beyond classic chronic PAD, including acute-on-chronic ischemia and selected non-occlusive vascular conditions; while this improves clinical generalizability, it also introduces heterogeneity that may complicate interpretation of calcification patterns across groups. Fifth, the LLAC scoring can be influenced by technical factors such as CT acquisition parameters and segment evaluability, particularly in distal vessels; through we used a standardized scoring workflow, measurement variability may persist. Formal inter-reader and intra-reader reproducibility metrics were not available, which limited confidence in the precision of segment-level LLAC measurements, particularly in smaller distal vessels. Finally, clinical outcome analyses (if included) were limited by short and heterogeneous follow-up and low event counts, and should be interpreted as exploratory. These constraints underscore the importance of further, more in-depth, forward-looking research to validate and amplify these results.

## 6. Conclusions

In this retrospective matched imaging-based study of patients with LEAD, overall LLAC scores did not differ consistently between DOAC users and non-anticoagulated controls when all exposure durations were analyzed together. However, exploratory duration-stratified analysis showed that patients with ≥5 years of continuous DOAC exposure had lower calcification scores in proximal segments, especially the infrarenal aorta and iliac arteries, while shorter exposure groups showed no consistent pattern; given the cross-sectional design, small subgroups, multiple comparisons, and possible residual confounding and survivor/healthy adherer bias, these findings should be considered strictly hypothesis-generating rather than evidence of a causal protective effect. No statistically robust reduction was seen in the crural arteries, where medial arterial calcification often predominates, suggesting that any association between DOAC exposure and calcification may be territory-specific, biology-specific, or masked by measurement variability and limited power in distal vessels. Gastrointestinal bleeding occurred only in the DOAC group and, although not statistically significant, and it represents a clinically relevant safety signal; therefore, these results should be viewed as a foundation for larger prospective multicenter longitudinal studies with baseline and follow-up imaging, predefined segment-level endpoints, careful adjustment for indications and preventive therapies, and mechanistic correlates to determine whether the observed long-term association reflects biology, clinical selection, or chance, and whether LLAC scoring may improve risk stratification and procedural planning in high-risk LEAD populations.

## Figures and Tables

**Table 1:** Summary of demographic data of the included 96 patients. DOAC—direct oral anticoagulant, IQR—interquartile range, LEAD—lower-extremity arterial disease, COPD—chronic obstructive pulmonary disease, ESRD—end-stage renal disease, GFR—glomerular filtration rate, AF—atrial fibrillation, DVT—deep venous thrombosis, PE—pulmonary embolism.

Variable	DOAC Group (*n* = 48)	Control Group (*n* = 48)
Age (years)	72.0 (median) IQR: 69.0–80.0	74.0 (median) IQR: 70.0–77.0
Sex, *n* (%)		
Male	37 (77%)	33 (69%)
Female	11 (23%)	15 (31%)
Indication of CT Scan, *n* (%)		
Aneurysmatic disease (iliac/popliteal)	4 (8%)	3 (6%)
Post-punction pseudoaneurysm/AV fistula	3 (6%)	3 (6%)
Chronic LEAD	34 (71%)	40 (83%)
Acute-on-chronic LE ischemia	7 (15%)	2 (4%)
Rutherford Classification (Chronic LEAD Cases Only), *n* (%-Relative to All Cases)		
Rutherford 1	1 (2%)	3 (6%)
Rutherford 2	11 (23%)	13 (27%)
Rutherford 3	11 (23%)	8 (17%)
Rutherford 4	1 (2%)	2 (4%)
Rutherford 5	5 (10%)	11 (23%)
Rutherford 6	5 (10%)	3 (6%)
Past Medical History, *n* (%)		
Smoking	23 (48%) *	29 (60%) *
Hypertension	45 (94%)	47 (98%)
Diabetes	25 (52%)	22 (46%)
Hyperlipidemia	33 (69%)	35 (73%)
Coronary artery disease	32 (67%)	27 (56%)
Cerebrovascular disease	8 (17%)	6 (13%)
Pulmonary disease (COPD)	8 (17%)	9 (19%)
Tumor	7 (15%)	7 (15%)
ESRD	6 (13%)	3 (6%)
Thyroid dysfunction	2 (4%)	4 (8%)
Concomitant medication use, *n* (%)		
Antiplatelet use	39 (81%)	46 (96%)
Statin use	39 (81%)	39 (81%)
DOAC Type/Dose (DOAC Group Only) *n* (%)		
Apixaban (Eliquis) 2 × 5 mg	15 (31%)	—
Apixaban (Eliquis) 2 × 2.5 mg	10 (21%)	—
Apixaban (Eliquis) 1 × 2.5 mg—GFR adjusted dose	2 (4%)	—
Rivaroxaban (Xarelto) 1 × 20 mg)	6 (13%)	—
Rivaroxaban (Xarelto) 1 × 15 mg	2 (4%)	—
Rivaroxaban (Xarelto) 1 × 10 mg—GFR adjusted dose	1 (2%)	—
Dabigatran (Pradaxa) 2 × 150 mg	6 (13%)	—
Dabigatran (Pradaxa or Telexer) 2 × 110 mg	2 (4%)	—
Edoxaban (Lixiana) 60 mg	2 (4%)	—
Edoxaban (Lixiana) 30 mg	2 (4%)	—
Reason for DOAC Use, (DOAC Group Only) *n* (%)		
AF	39 (81%)	—
DVT	2 (4%)	—
PE	2 (4%)	—
DVT + PE	1 (2%)	—
Previous stroke	1 (2%)	—

* No data regarding smoking habits is available in 12 patients from DOAC, and 8 patients from the control group.

**Table 2:** Perioperative and follow-up data of the 96 included patients. DOAC—direct oral anticoagulant, GI—gastrointestinal, LE—lower-extremity.

Variable	Control Group (*n* = 48)	DOAC Group (*n* = 48)	*p*-Value
Treatment after CT			
Medical therapy	12 (25%)	9 (19%)	0.621
Endovascular	14 (29%)	14 (29%)	1.000
Open surgery	9 (19%)	14 (29%)	0.339
Hybrid procedure	9 (19%)	5 (10%)	0.386
No revascularization option	4 (8%)	6 (13%)	0.740
Segments Treated			
Aorto-iliac	1 (2%)	2 (4%)	1.000
Aorto-iliac + femoro-popliteal	1 (2%)	-	1.000
Iliac alone	7 (15%)	9 (19%)	0.784
Iliac + femoro-popliteal	9 (19%)	4 (8%)	0.232
Femoro-popliteal alone	13 (27%)	15 (31%)	0.822
Crural alone	-	1 (2%)	1.000
Femoro-popliteal + crural	1 (2%)	2 (4%)	1.000
Reoperations			
Patients with 1 reoperation	6 (13%)	8 (17%)	0.772
Patients with >1 reoperations	5 (10%)	2 (4%)	0.435
Total number of reoperations	16 (33%)	13 (27%)	0.657
Timing of Reoperation			
Reoperation < 30 days	4	9	0.232
Within 24 h	3	3	1.000
>30 days	12	4	0.053
Amputations			
Total procedure number	8 (17%)	8 (17%)	1.000
<30 days	3	5	0.714
>30 days (≤6 months)	5	3	0.714
Femoral	2	6	0.268
Crural	1	0	1.000
Below ankle	5	2	0.435
Other Adverse Events			
Acute myocardial infarction	6 (13%)	4 (8%)	0.740
Ischemic stroke	1 (2%)	1 (2%)	1.000
Bleeding (GI)	0	5 (10%)	0.056
Bleeding (nasal)	0	2 (4%)	0.495
Hemorrhagic stroke	0	1 (2%)	1.000
Mortality	6 (13%)	9 (19%)	0.574
Causes of Death			
Complications due to metastatic cancer	1	-	
Perforated cholecystitis	-	1	
Pneumonia	1	0	
Cardiovascular	2	2	
Sepsis from LE wound	1	4	
Stroke (ischemic)	1	1	
Suicide	0	1	

Values are *n* (%) unless otherwise specified.

**Table 3:** Comparison of LLAC scores. LLAC—lower-extremity arterial calcification, DOAC—direct oral anticoagulant, IQR—interquartile range.

Segment Type	DOAC Median (LLAC/mm)	Control Median (LLAC/mm)	*p*-Value	Interpretation
Infrarenal aorta	7596.0 (IQR: 5063.0–11,712.0)	8637.0 (IQR: 5626.0–12,241.0)	0.487	No significant difference
Total iliac	5689.5 (IQR: 2748.1–7773.9)	5193.5 (IQR: 3933.5–8919.5)	0.602	No significant difference
Total fem-pop	6089.0 (IQR: 3522.0–13,311.8)	6582.0 (IQR: 4203.5–11,344.0)	0.789	No significant difference
Total crural	1349.5 (IQR: 531.3–3114.5)	1431.5 (IQR: 814.9–3681.0)	0.390	No significant difference

**Table 4:** Duration-stratified unpaired comparison of LLAC between DOAC users and matched controls. Values are reported as median (IQR) for each arterial region within DOAC duration strata (1–2 y, 3–4 y, ≥5 y). *p*-values are from two-sided paired Wilcoxon signed-rank tests, with significance defined as *p* < 0.05 (exploratory).

Duration Stratum	Segment	DOAC Median LLAC (IQR)	Control Median LLAC (IQR)	*p*-Value	Interpretation
1–2 y	Infrarenal aorta	8838.5 (5219.2–12,423.0)	6098.5 (3960.8–11,682.2)	0.312	No significant difference
1–2 y	Total iliac	13,582.5 (4801.5–15,631.5)	8006.0 (5706.2–10,625.8)	0.260	No significant difference
1–2 y	Total femoro-popliteal	16,613.5 (9789.8–29,065.8)	9308.5 (7470.0–13,373.8)	0.126	No significant difference
1–2 y	Total crural	3782.5 (1162.0–7828.2)	2024.5 (1438.5–3628.2)	0.707	No significant difference
3–4 y	Infrarenal aorta	10,570.0 (7475.0–14,353.0)	8269.0 (5085.8–10,268.0)	0.139	No significant difference
3–4 y	Total iliac	10,905.5 (6850.2–14,778.2)	7524.5 (4570.0–16,567.0)	0.395	No significant difference
3–4 y	Total femoro-popliteal	11,515.0 (710.8–23,424.8)	5888.5 (2897.0–17,440.8)	0.982	No significant difference
3–4 y	Total crural	4030.0 (1176.0–6106.2)	2554.5 (1180.5–8096.2)	0.945	No significant difference
≥5 y	Infrarenal aorta	5593.5 (4521.2–8565.8)	11,185.0 (6973.0–14,395.0)	0.001997	Significant difference
≥5 y	Total iliac	5624.5 (3012.0–7812.5)	11,501.0 (8684.8–19,352.8)	0.001867	Significant difference
≥5 y	Total femoro-popliteal	7809.0 (4565.2–12,814.0)	18,827.0 (10,591.2–22,566.2)	0.0557	No significant difference (trend only)
≥5 y	Total crural	1839.5 (990.8–5612.8)	4793.5 (1944.5–8414.2)	0.0588	No significant difference (trend only)

## Data Availability

The de-identified data analyzed during the current study are not publicly available due to institutional policies and ethics restrictions, but may be shared by the corresponding authors on reasonable request and with approval from the institutional ethics board.

## Abbreviations

The following abbreviations are used in this manuscript:

Abbreviation	Definition
ADMIRE	Advanced Modeled Iterative Reconstruction
AF	atrial fibrillation
CKD	chronic kidney disease
COPD	chronic obstructive pulmonary disease
CT	computed tomography
CTA	computed tomography angiography
DM	diabetes mellitus
DOAC	direct oral anticoagulant
DVT	deep venous thrombosis
ESRD	end-stage renal disease
FP	femoro-popliteal
GFR	glomerular filtration rate
GI	gastrointestinal
HU	Hounsfield units
IQR	interquartile range
LE	lower-extremity
LEAD	lower-extremity arterial disease
LLAC	lower limb arterial calcification
MAC	medial arterial calcification
MACE	major adverse cardiovascular events
MGP	matrix Gla protein
PAD	peripheral artery disease
PE	pulmonary embolism
SPSS	Statistical Package for the Social Sciences
VKA	vitamin K antagonist
VSMC	vascular smooth muscle cell
VTE	venous thromboembolism
